# The 2020 to 2021 California megafires and their impacts on wildlife habitat

**DOI:** 10.1073/pnas.2312909120

**Published:** 2023-11-20

**Authors:** Jessalyn Ayars, H. Anu Kramer, Gavin M. Jones

**Affiliations:** ^a^United States Department of Agriculture Forest Service, Rocky Mountain Research Station, Albuquerque, NM 87102; ^b^Biology Department, University of New Mexico, Albuquerque, NM 87131; ^c^Department of Forest and Wildlife Ecology, University of Wisconsin, Madison, WI 53706

**Keywords:** climate change, fire severity, megafire, wildfire, wildlife habitat

## Abstract

Fire activity during 2020 to 2021 in California, USA, was unprecedented in the modern record. More than 19,000 km^2^ of forest vegetation burned (10× more than the historical average), potentially affecting the habitat of 508 vertebrate species. Of the >9,000 km^2^ that burned at high severity, 89% occurred in large patches that exceeded historical estimates of maximum high-severity patch size. In this 2-y period, 100 vertebrate species experienced fire across >10% of their geographic range, 16 of which were species of conservation concern. These 100 species experienced high-severity fire across 5 to 14% of their ranges, underscoring potentially important changes to habitat structure. Species in this region are not adapted to high-severity megafires. Management actions, such as prescribed fires and mechanical thinning, can curb severe fire behavior and reduce the potential negative impacts of uncharacteristic fires on wildlife.

Fire is an essential forest disturbance, creating heterogeneity in forest structure that generates wildlife habitat. However, intensification of wildfire driven by climate and land-use change threatens the very existence of forests (1) and more than 4,400 vertebrate species (2, 3). Fires across the United States were four times larger, three times as frequent, and more widespread from 2000 to 2019 than from 1985 to 1999 (4). In California, USA, 2020 and 2021 were unusually intense wildfire years: While few individual fires were outside the size range of the past 100 y, the number of large fires and overall area burned in 2020 and 2021 was unprecedented (5, 6), and was primarily driven by large, severe fires in the dry, mountainous Western Cordillera ecoregion (Fig. 1). In conifer forests in California, patches of high-severity fire have been increasing in size and regularity of shape (7). Additionally, megafires (>40,000 ha) burned more area in shrubland than conifer forests from 2000 to 2020 (8). However, we lack basic knowledge of the impacts of these fires on wildlife habitats on a broad scale.

**Fig. 1.**
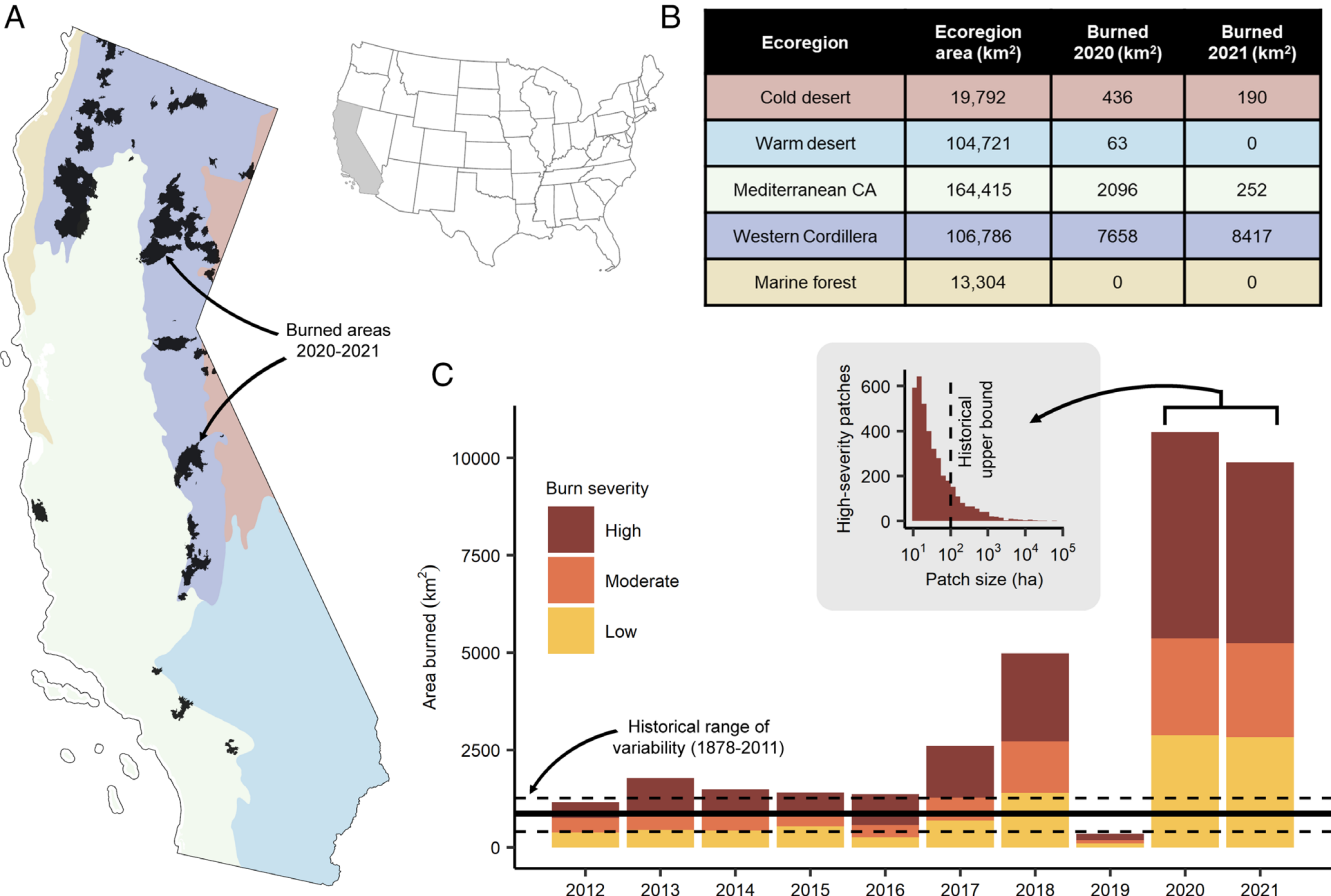
Wildfire distribution in California (USA). (*A*) Dark shading indicates 2020 and 2021 wildfires. Colored regions denote ecoregions, which are defined in *B*. A map of the continental United States with California highlighted is provided for context. (*B*) Square kilometers burned in the 2020 and 2021 wildfire seasons by ecoregion. (*C*) Annual area burned (2012 to 2021) in California by CBI4 fire severity class. Horizontal black lines represent the 25th (lower dashed line), 50th (middle solid line), and 75th (upper dashed line) quantiles of annual area burned in California from 1878 to 2011, representing a historical range of variability. The *Inset* histogram shows the distribution of 2020 to 2021 high-severity patch sizes in California compared to the estimated historical upper bound (black vertical dashed line). There was some overlap in fire footprints between 2020 and 2021 as well as across 2012 to 2021.

We quantified the amount and quality of wildlife habitat burned in the 2020 and 2021 wildfire seasons in California, USA, by overlaying habitat suitability models for vertebrate species in California with a four-class fire severity index (CBI4). We acquired area burned and average habitat suitability within each fire severity class in each species’ range, and then identified patterns by fire severity, conservation status, and taxonomic group (amphibians, birds, mammals, and reptiles).

## Results

During 2020 and 2021, 19,090 km^2^ burned in California, of which 84% (16,069 km^2^) burned in the Western Cordillera (Fig. 1). These 2 y account for 58% of the area in California that burned from 2012 to 2021, and in each year, the total area burned was >10× greater than the historical average from 1878 to 2011. In 2020 to 2021, 701 high-severity patches exceeded the estimated historical maximum high-severity patch size (~100 ha) (7). These large patches made up 89% of the area burned at high severity in 2020 to 2021 (Fig. 1).

**Fig. 2.**
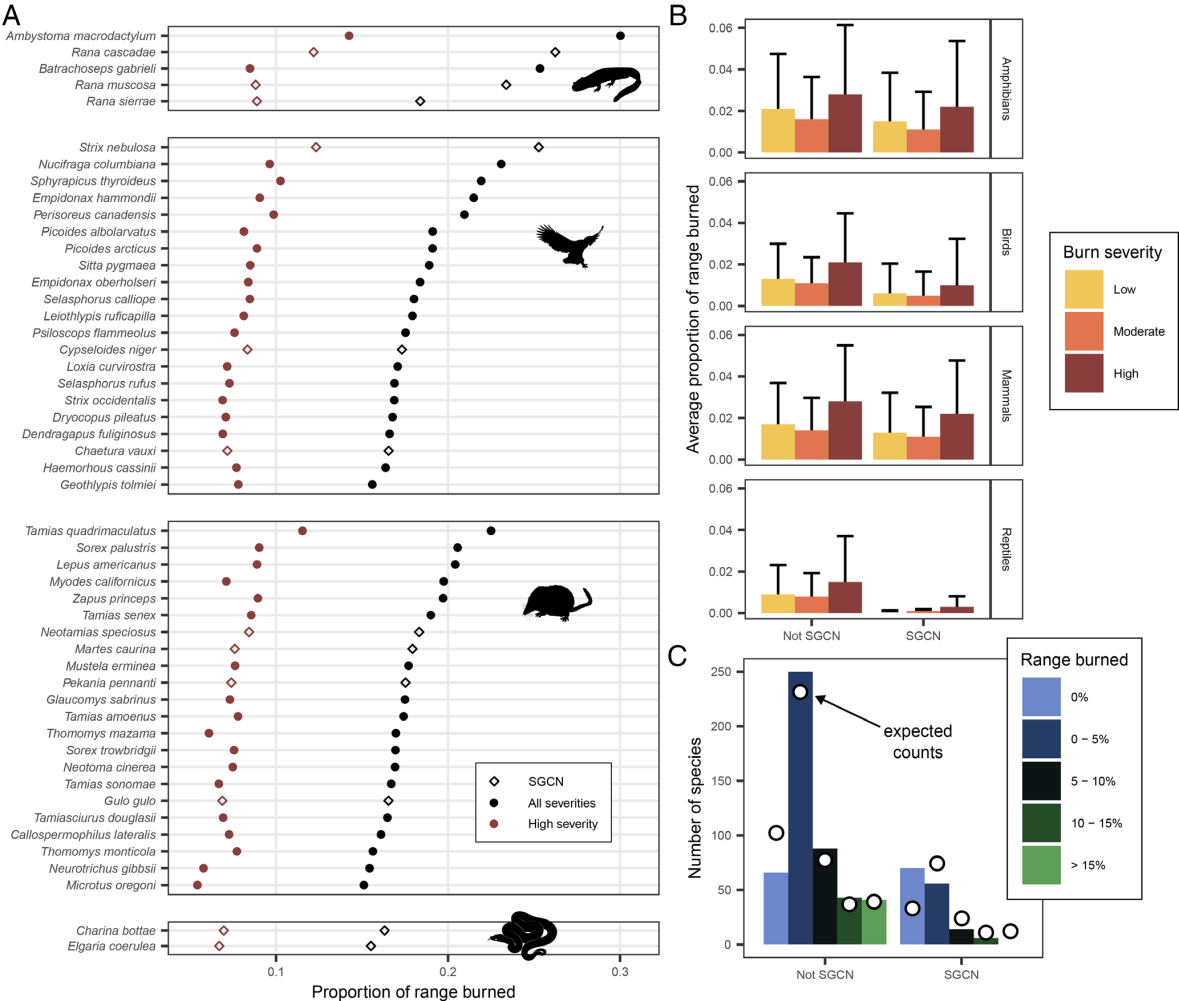
Quantity and quality of habitat burned in 2020 and 2021 (combined). (*A*) The 50 species with the highest proportion of their range burned at all fire severities, by taxon. The black series indicates the proportion of range burned at all fire severities, and the red series indicates proportion of range burned at high severity. Open diamonds represent California SGCN (hereafter special-status species). (*B*) Average proportion of range burned for special-status versus other species by taxa. Error bars indicate SE. Area burned was different between special-status and other birds and reptiles for all burn severities (*P* < 0.001), but not between special-status and other amphibians or mammals. (*C*) Number of special-status and other species by percent of habitat burned. Dots indicate the expected counts based on number of species in range burned categories. Observed counts were significantly different than expected (χ^2^_4_ = 71.4, *P* < 0.001).

One hundred vertebrate species experienced fire across >10% of their range. Fifty of these experienced fire across 15 to 30% of their ranges, with 5 to 14% of their ranges burning at high severity (Fig. 2*A*). More species of the greatest conservation need (SGCN) (hereafter, “special-status species”; see *SI Appendix*) than expected experienced no burning in their ranges, and fewer special-status species than expected had any amount of their range burn (χ^2^_4_ = 71.4, *P* < 0.001, Fig. 2*C*). These patterns were driven by birds and reptiles: There was more unburned area and less burned area for special-status species in these groups compared to other species (all *P* ≤ 0.001, Fig. 2*B*). The suitability of habitat burned did not differ between special status and other species (by fire severity for any taxon, all *P* > 0.10).

## Discussion

One-hundred vertebrate species experienced burning over >10% of their ranges. Five to fourteen percent of these species’ ranges burned at high severity. High-severity fire in 2020 to 2021 tended to occur in large contiguous patches (Fig. 1); 16 high-severity patches exceeded 10,000 ha. High-severity patches of this size have no historical analog in California forests and have negative population consequences for sensitive vertebrate species and animal communities (9).

Special-status species were not disproportionately affected by the 2020 and 2021 California megafires, and birds and reptiles of greatest conservation need had less of their range burn than expected, even at high severity. While this is encouraging, the overall area burned and at high severity was large for all species, and the effects of fire on special-status species should be addressed on a case-by-case basis. The long-toed salamander (*Ambystoma macrodactylum*), which experienced high-severity fire across a greater portion of its range than any other species examined (14%), has been shown to decline 1 to 2 decades postfire, particularly in the most severely burned areas (10). The most fire-affected bird, the great gray owl (*Strix nebulosa*), has been shown to persist immediately after severe fires and is thus thought to be fire-resilient (11). But, for this and other species examined here, there is simply no frame of reference for understanding population responses to the scale and severity of the fires seen in 2020 to 2021 in California, and it is unlikely that many species have adaptations to survive these fire events (12). While our goal was to examine broad-scale fire impacts on habitat, species’ responses to wildfire are highly variable and will depend on life-history and other traits that should be a focus of future research (13).

Wildfire does not inherently destroy wildlife habitat: Natural fire regimes create spatiotemporal habitat heterogeneity that supports biodiversity (14). However, when fire regimes rapidly change, wildlife may be unable to respond appropriately (3). Unusually severe fire, coupled with shorter fire return intervals, may result in ecosystem state changes from forest to shrubland or grassland (1). Additionally, other stressors exacerbated by climate change (i.e., drought, invasive species) limit forest resilience to disturbance. In the face of these pressures, ecosystem restoration treatments including prescribed fires, managed wildfires, and mechanical thinning have the potential to improve forest resilience (15). The large amount of wildlife habitat that burned in 2020 and 2021 at high severity, coupled with the likelihood that extreme wildfire seasons will be more common in the future, underlines the importance of increasing the pace and scale of dry forest ecosystem restoration.

## Materials and Methods

To describe wildfire severity and coverage, we used the four-class standardized composite burn index (CBI4) from the Rapid Assessment of Vegetation Condition After Wildfire Program (RAVG). We used the Fire and Resource Assessment Program database to compute average area burned from 1878 to 2011. We used the California Wildlife Habitat Relationships system to obtain spatial data on habitat suitability for terrestrial vertebrates in California.

We overlaid species habitat suitability maps on RAVG maps for 2020 and 2021 (combined) to determine the area and the average suitability of habitat burned at each fire severity class for each species. We conducted Student’s *t* tests to evaluate differences in means of i) suitability of habitat burned and ii) the average proportion of range burned between special-status and other species across taxa and burn severity. We conducted a Chi-squared test to determine whether the distribution of special-status species differed across bins of proportion of range burned from expectations. Full methods are described in *SI Appendix*.

## Supplementary Material

Appendix 01 (PDF)

## Data Availability

Code used in the analysis have been deposited in GitHub (https://github.com/jayars99/megafires-20-21-public). All other data are included in the manuscript and/or *SI Appendix*.
