# Supplementary material for: The 2020 to 2021 California megafires and their impacts on wildlife habitat
Source: Proc Natl Acad Sci U S A. 2023 Nov 20;120(48):e2312909120. doi: 10.1073/pnas.2312909120 (PMC10691208; doi:10.1073/pnas.2312909120)
Supplement: Supplementary file 1 — Appendix 01 (PDF) [file pnas.2312909120.sapp.pdf]

SI Appendix

Materials and Methods

Fire extent and severity
To describe wildfire severity and coverage, we used the four-class standardized composite burn index (CBI4) from the Rapid Assessment of Vegetation Condition After Wildfire Program (RAVG) provided by the USDA Forest Service Geospatial Technology and Applications Center (GTAC). RAVG data is produced for wildland fires that burned at least 1,000 acres of forested National Forest Service (NFS) lands across the United States and provides a robust estimate of fire activity more rapidly than other data options (https://burnseverity.cr.usgs.gov/products/ravg). Data is available for 2012 to present, summarized annually. RAVG calculates the relative differenced normalized burn ratio (RdNBR) for before and 30-45 days after the wildfire using primarily Landsat imagery, and also Sentinel 2 imagery for some fires beginning in 2019 (all data is presented at 30-meter resolution). Regression equations based on tree mortality field data are used to relate RdNBR to CBI4. CBI4 classes are defined as unchanged, low severity, moderate severity, and high severity. We equated the unchanged category with the unburned category outside of fire footprints. We also created a layer of 2020 and 2021 fires, where fire severity class was the higher of the two years in overlapping fires, to summarize wildlife habitat burned over 2020 and 2021. RAVG is known to occasionally overestimate area burnt at high severity due to ash left on the soil immediately after fire; however, it is the best data available for recent fires. We also computed patch size of areas burned at high severity for patches of at least 10 ha. Processing of spatial data was performed using R (version 4.3.0), spatial analysis packages raster, sf, stars, and terra, and the University of Wisconsin high throughput computing system (HTCondor, https://htcondor.org/).

Longer-term fire severity summary
To provide longer-term context to the distribution of wildfire severity in California, we calculated the area of forested NFS lands that burned at each severity class from 2012-2021 within the state of California, divided by the Commission for Environmental Cooperation's Level II ecoregions, which are useful for subcontinental overviews of ecological patterns. For each year, we computed the area (km^2) in each burn severity category. We also used the Fire and Resource Assessment Program database (FRAP, fire.ca.gov) to compute area burned from 1878-2011.

Vertebrate species habitat suitability
We used the California Wildlife Habitat Relationships (CWHR) system to obtain spatial data on habitat suitability for vertebrate species (amphibians, birds, mammals, and reptiles) in California. The CWHR models were developed for the 2015 CA State Wildlife Action Plan (SWAP), which is updated every ten years. The CHWR database contains life history, geographic range, and suitability, and whether the species is designated as California species of greatest conservation need (SGCN; hereafter, 'special-status species') for 712 species of amphibians, birds, fish, mammals, and reptiles in California at 30-m resolution. The SGCN or special-status designation is given to species which are federally listed under the Endangered Species Act, are a California species of special concern, or are climate vulnerable according to the SWAP. It also maps 59 generalized habitat types across the state. Models of range and habitat suitability are generated by surveying experts on the species' range and preference for each habitat type, and then relating survey responses back to the state habitat map using a community-level matrix model. We filtered out fish, marine animals, and non-native species, masked out bodies of water and the ocean, transformed the data to match the RAVG projection, and resampled it to match the RAVG grid.

Summarizing wildlife habitat exposed to wildfire
We overlaid species habitat suitability maps on RAVG maps for each Level II ecoregion for 2020 and 2021 (combined) to determine the area of habitat burned at each fire severity class for each species, and the average suitability of habitat burned at each fire severity class for each species.

We quantified the proportion of habitat burned at each fire severity class and the average suitability of habitat burned at each fire severity and by ecoregion, taxonomic group, and special-status. We conducted Student's *t*-tests to evaluate potential differences in means of (*i*) suitability of habitat burned, and (*ii*) the average proportion of range burned between special-status and other species across

taxa and burn severity. To conduct this test, data must be continuous, variance must be similar among groups, must be normally distributed; all these assumptions were met. We also conducted a Chi-squared test to determine whether the distribution of special-status species within bins of proportion of range burned (0, 0-5, 5-10, 10-15, and >15%) was different than expected. Data used in Chi-squared tests must be independent and mutually exclusive; these assumptions were met.

All data used in this study is publicly available via the Rapid Assessment of Vegetation Condition after Wildfire (RAVG) database and the California Wildlife Habitat Relationships (CWHR) database. Code used in the analysis are available at github.com/jayars99/megafires-20-21-public.
